# Safflor Yellow B Attenuates Ischemic Brain Injury via Downregulation of Long Noncoding AK046177 and Inhibition of MicroRNA-134 Expression in Rats

**DOI:** 10.1155/2020/4586839

**Published:** 2020-06-03

**Authors:** Chaoyun Wang, Hongzhi Wan, Qiaoyun Wang, Hongliu Sun, Yeying Sun, Kexin Wang, Chunxiang Zhang

**Affiliations:** ^1^Hearing and Speech Institute, Binzhou Medical University, Yantai 264003, China; ^2^School of Pharmaceutical Sciences, Binzhou Medical University, Yantai 264003, China; ^3^Third Class of Senior High School, No. 2 Middle School of Yantai Shandong, Yantai, China; ^4^Department of Biomedical Engineering, School of Medicine, University of Alabama at Birmingham, Birmingham, AL, USA

## Abstract

Stroke breaks the oxidative balance in the body and causes extra reactive oxygen species (ROS) generation, leading to oxidative stress damage. Long noncoding RNAs (lncRNAs) and microRNAs play pivotal roles in oxidative stress-mediated brain injury. Safflor yellow B (SYB) was able to effectively reduce ischemia-mediated brain damage by increasing antioxidant capacity and inhibiting cell apoptosis. In this study, we investigated the putative involvement of lncRNA AK046177 and microRNA-134 (miR-134) regulation in SYB against ischemia/reperfusion- (I/R-) induced neuronal injury. I/R and oxygen-glucose deprivation/reoxygenation (OGD/R) were established *in vivo* and *in vitro*. Cerebral infarct volume, neuronal apoptosis, and protein expression were detected. The effects of SYB on cell activity, cell respiration, nuclear factor erythroid 2-related factor 2 (Nrf2), antioxidant enzymes, and ROS were evaluated. I/R or OGD/R upregulated the expression of AK046177 and miR-134 and subsequently inhibited the activation and expression of CREB, which caused ROS generation and brain/cell injury. SYB attenuated the effects of AK046177, inhibited miR-134 expression, and promoted CREB activation, which in turn promoted Nrf2 expression, and then increased antioxidant capacities, improved cell respiration, and reduced apoptosis. We suggested that the antioxidant effects of SYB were driven by an AK046177/miR-134/CREB-dependent mechanism that inhibited this pathway, and that SYB has potential use in reducing or possibly preventing I/R-induced neuronal injury.

## 1. Introduction

Stroke is an important cerebrovascular disease that afflicts many people worldwide and frequently causes death or long-term disability [1]. Ischemic stroke is the most common type, accounting for about 80% of all strokes [2, 3]. Brain injury is caused by disruption of blood flow to the brain and is characterized by oxidative stress. In addition, reoxygenation resulting from the restoration of blood flow exacerbates tissue damage [4].

Pathophysiologically, ischemia and reperfusion can inhibit the activity of endogenous antioxidant enzymes and promote the overproduction of reactive oxygen species (ROS) [5–7]. Previous studies have shown that antioxidants significantly reduce ischemic damage through the inhibition of ROS production [8–11]. Nuclear factor erythroid 2-related factor 2 (Nrf2) is a reduction-oxidation- (redox-) sensitive transcription factor that binds to antioxidant response elements (ARE) and activates the transcription of antioxidant enzymes. Studies have shown that cysteine residues on protein Keap1 are oxidized by ROS, leading to the release and activation of Nrf2 [12, 13]. Thus, Nrf2 is a useful therapeutic target for reducing or preventing ROS damage in the brain following ischemia/reperfusion (I/R) injury. Cyclic AMP (cAMP) response element-binding protein (CREB) is a leucine zipper transcription factor that inhibits ROS generation and suppresses severe ischemic injury by upregulating brain-derived neurotrophic factor (BDNF) and Bcl-2 [14, 15].

MicroRNAs (miRNAs) are endogenous, short (≈22 nucleotides), noncoding single-strand RNAs that regulate gene expression at the posttranscriptional level by influencing the translation of specific target mRNAs. Recent research revealed that a variety of miRNAs play important roles in ischemic injury through the modulation of cellular redox reactions and mitochondrial function [16]. For example, downregulation of miR-134 enhances Bcl-2 expression and alleviates ischemic injury by regulating CREB activity [17].

Long noncoding RNAs (lncRNAs) play key roles in various cellular contexts under both physiological and pathological conditions, and they are involved in diverse biological processes such as RNA processing, modulation of apoptosis and invasion, and chromatin modification [18–20]. AK046177 is a 606-base pair (bp) noncoding RNA sequence derived from a gene sequence (from 116850844 to 116851448) located on chromosome 13.

**Figure 1:**
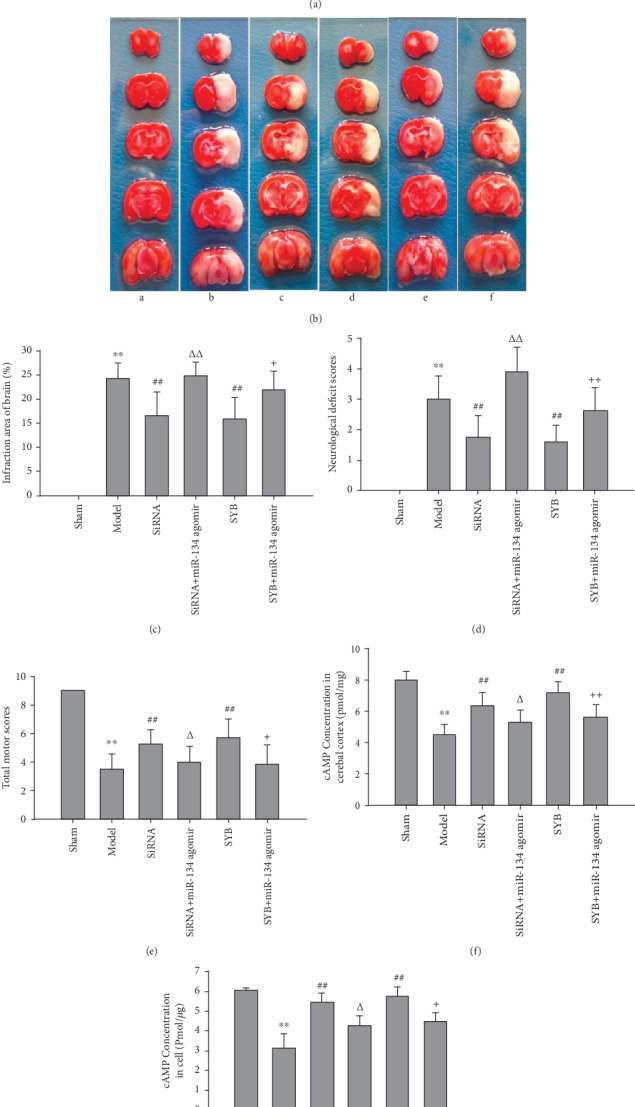
Effect of safflor yellow B on neurological deficit score, infarction area, total motor score, and cAMP level. (a) Chemical structure of safflor yellow B. As shown in (b)–(e), rats were divided into six groups: sham, ischemia/reperfusion (I/R), AK046177 siRNA, AK046177 siRNA+miR-134 agomir, SYB, SYB+miR-134 agomir. Except for the sham group, all rats in the other groups had established ischemia for 1 h followed by reperfusion for 23 h. SYB and saline were administrated by tail vein continuously for three days before treatment with I/R, and miR-134 agomir and AK046177 siRNA were given via intracerebroventricular injection. The neurological deficit score of each rat was obtained according to Longa's method. Infarct volumes were measured by staining brain sections with 2,3,5-triphenyltetrazolium chloride. (a) represents pathological changes of cerebral infarction ((a) sham; (b) I/R; (c) AK046177 siRNA; (d) AK046177 siRNA+miR-134 agomir; (e) SYB; (f) SYB+miR-134 agomir). (b) represents neurological deficit scores. (c) represents the infarction area. (d) represents total motor scores (*n* = 8). As shown in (f)–(g), cAMP levels in the cerebral cortex and primary fetal cortical cells were detected using a ^125^I-radioimmunoassay according to the method described by the manufacturer. Data are presented as mean ± S.D. (*n* = 8 in tissues; *n* = 3 in cells). One-way ANOVA test was used to determine statistical significance. ^∗∗^*P* < 0.01*vs.* the sham group or the control group, ^##^*P* < 0.01*vs.* the I/R group or the OGD/R group, ^Δ^*P* < 0.05 or ^ΔΔ^*P* < 0.01*vs.* the AK046177 siRNA group, and ^+^*P* < 0.05 or ^++^*P* < 0.01*vs.* the SYB group.

Safflower yellow is the flavonoid compound extracted from *Carthamus tinctorius* L., which includes the components hydroxysafflor yellow A (HSYA) and safflor yellow B (SYB). It has been shown to effectively reduce oxidative stress-mediated damage [21, 22]. A study by Wang et al. demonstrated that HSYA significantly increases antioxidant enzyme activity by activating the cAMP/PKA signaling pathway [23]. SYB (Figure 1(a)) is a yellow amorphous powder with a purity of more than 98% by HPLC, and it is water soluble and has demonstrated protective effects in neuronal injury models induced by oxidative stress [24, 25]. However, its effect on brain injury induced by I/R remains to be investigated. This study tested whether SYB reduces I/R-mediated brain injury, and evaluated its potential mechanisms by studying changes in the expression of AK046177, miR-134, Nrf2, and CREB.

## 2. Materials and Methods

### 2.1. Experimental Design and Cerebral I/R

Male Sprague-Dawley rats (11 months of age), weighing about 350 to 400 g, were purchased from the Experimental Animal Department of Shandong Luye Pharmaceutical Co. Ltd. (Yantai, China). All animals (5 rats per cage) were bred in a temperature-controlled animal facility with a 12 h light/dark cycle.

Animals were randomly assigned to the following 6 groups: sham (*n* = 19), I/R (*n* = 19), I/R+AK046177 siRNA oligo (*n* = 19), I/R+SYB (*n* = 19), I/R+SYB+miR-134 agomir (*n* = 19), and I/R+AK046177 siRNA oligo+miR-134 agomir (*n* = 19).

Rats were anesthetized with 10% chloral hydrate in 0.9% NaCl (300 mg/kg, i.p.) and placed on a 37°C temperature-controlled heating pad. AK046177 siRNAs and miR-134 agomir were synthesized by GenePharma (Shanghai, China). Except for the sham group, all rats from the other groups were treated as follows: every morning at 9 o'clock, 2 ml of SYB or saline was administered intravenously at a dose of 6 mg/kg continuously for 3 days prior to I/R injury, and 4 *μ*l of AK046177 siRNA oligo (100 nM) or miR-134 agomir (80 nM) with Lipofectamine was administered via intracerebroventricular infusion half an hour before ischemia

Middle cerebral artery occlusion (MCAO) was performed according to the monofilament method as described by Longa et al. and Macrae [26, 27]. After 1 h of ischemia followed by 23 h of reperfusion, a single experimenter blinded to treatment condition determined the neurological deficit score of each rat according to Longa et al.'s previously validated five-point scale, described below [26]. Following neurological evaluation, rats were decapitated under deep anesthesia and brains were removed. Some whole brains were sectioned and infarct volume was measured by staining with 2,3,5-triphenyl-tetrazolium chloride (TTC; 1.5%). The right part from the same region of the cerebral cortex (the damaged hemisphere for MCAO) and other brain tissues were isolated and stored at −80°C in a freezer. All animals were treated in accordance with the National Institutes of Health Guide for Care and Use of Laboratory Animals (NIH Publications No. 8023, revised 1996). Animal care and experimental procedures were approved by the Ethics Committee on Animal and Human Experimentation of Binzhou Medical University (approval no. 2016077).

### 2.2. Neurological Tests

After 23 hours of reperfusion, eight rats of each group were subjected to a modified neurological examination designed to evaluate total motor deficit [28]. Briefly, rats were placed on a 10-20 cm horizontal screen, which was rotated from a horizontal to vertical position. The length of time each rat remained on the vertical screen was recorded and scored as 1 point per 5 s, to a maximum of 15 s (3 points). Rats were then placed at the center of a horizontal wooden rod, and the length of time they remained on the rod was recorded and scored as 1 point per 10 s, to a maximum of 30 s (3 points). Rats were placed on a horizontal rope, and the length of time each rat remained on the rope was recorded and scored as 1 point per 2 seconds, to a maximum of 6 s (3 points). A total motor score (TMS) was calculated based on the results of these assessments (maximum 9 points).

### 2.3. TUNEL Staining

After 23 hours of reperfusion, three rats of every group were anesthetized and intracardially perfused with 4% paraformaldehyde (PFA) in 0.1 M phosphate-buffered saline (PBS; pH 7.4). Following perfusion, the brains were removed and postfixed overnight in 4% PFA at 4°C. The brains were embedded in paraffin and sliced into coronal sections (5 *μ*m; Leica Biosystems, Wetzlar, Germany). Apoptosis was detected using an *in situ* cell death detection kit (Roche, Germany), in accordance with the manufacturer's protocol. Samples were stained with DAPI for 5 min, following which apoptotic (TUNEL-positive) cells were visualized as localized bright red signals on a black background using a DMR fluorescence microscope (Leica Microsystems, Wetzlar, Germany). An apoptotic index (AI) was determined as the ratio of apoptotic cells to the total number of cells, averaged from 3 sections per animal.

### 2.4. Cell Culture

Primary cortical cells from the cerebral cortex of fetal rats (E16–18) were grown in Dulbecco's Minimal Essential Medium (DMEM) supplemented with 10% (*v*/*v*) fetal bovine serum, 1% penicillin/streptomycin, and 3.7 g/l NaHCO_3_. Before the initiation of the experiment, the cells were seeded in dishes and precultured for 3 to 5 days at 37°C in a humidified incubator, with 5% CO_2_ and 95% air. They were then cultured and divided into 7 groups: control, oxygen-glucose deprivation/reoxygenation (OGD/R), AK046177 siRNA (OGD/R+siRNA), AK046177 siRNA+agomiR-134 (OGD/R+siRNA+agomiR-134), SYB (OGD/R+SYB), SYB+agomiR-134 (OGD/R+SYB+agomiR-134), and negative control (OGD/R+NC). All groups excluding the control group were cultured in glucose-free DMEM under hypoxic conditions (1% O_2_/94% N_2_/5% CO_2_) at 37°C for 4 h. Thereafter, media in all groups was replaced with normal DMEM, and culturing continued for 20 h of reoxygenation under normoxic conditions (95% air/5% CO_2_). Cells were pretreated with SYB (final concentration of 0.5 mmol/l), AK046177 siRNA (80 nmol/l), and agomiR-134 (50 nmol/l) for 24 h prior to OGD/R. siRNA, agomiR-134, or control antagomir were transfected into primary cortical cells for 24 h using Lipofectamine RNAiMAX Transfection Reagent (Invitrogen) according to the manufacturer's protocol.

### 2.5. Assessment of Cell Viability

The 3-(4,5-dimethyithiazol-2-yl)-2,5-diphenyl-tetrazolium bromide (MTT) assay was used to determine cellular mitochondrial dehydrogenase activity in primary cortical cells (2 × 10^4^/ml) cultured in 96-well plates. Dark blue formazan crystals formed in intact cells which were solubilized with dimethyl sulfoxide (DMSO). The absorbance was measured at 490 nm with a microplate reader (Thermo Fisher Scientific, Waltham, MA, USA). Results were expressed as the percentage of MTT reduced, and data were normalized to the absorbance of control cells, considered 100%.

### 2.6. Measurement of Apoptosis

Primary cortical cells (9 × 10^5^/ml) cultured in 6-well plates were harvested and processed in accordance with the procedure of the Annexin V-FITC Apoptosis Detection Kit (BD Biosciences). Finally, flow cytometry (Epics-XL, Beckman Coulter, USA) was performed to quantify apoptosis. The results are expressed as percent of the control value.

### 2.7. Measurement of Antioxidative Enzyme Activity and Malondialdehyde Level

In vivo cerebral cortical tissue was dissected and homogenized, and in vitro cells (4 × 10^5^/ml) were harvested and lysed using ultrasound, then centrifuged at 12,000 g for 5 min at 4°C. The supernatants were collected, and the activities of superoxide dismutase (SOD) and glutathione peroxidase (GPx), as well as the malondialdehyde (MDA) concentration, were detected according to the manufacturer's assay kits (Nanjing Jiancheng Bio-Engineering Institute Co., Ltd.).

### 2.8. Assay of Intracellular Total ROS Levels

Intracellular ROS production in primary cells was assessed by measuring the fluorescence intensity of 2,7-dichlorodihydrofluorescein diacetate (DCFH-DA). After being reoxygenated for 20 h, cells (9 × 10^5^/ml) were treated with 10 *μ*M DCFH-DA in PBS in the dark at 37°C for 30 min, and then washed with PBS to remove excess dye. The level of fluorescence intensity was immediately evaluated at (excitation) 488 nm and (emission) 525 nm with a microplate reader (BioTek Synergy H4, USA).

### 2.9. Measurement of Intracellular cAMP Concentration

Primary cortical neurons were seeded in 6-well plates, and cells (1 × 10^6^/ml) were subjected to OGD/R, then harvested by centrifugation at 1000 g for 10 min at 4°C. Cerebral cortical tissue and cells were homogenized in 50 mmol/l acetic acid buffer (pH 4.75) and lysed by sonication. The homogenate was centrifuged at 3000 g for 15 min at 4°C. cAMP levels in the supernatant were determined using a ^125^I-radioimmunoassay according to the RIA kit instructions (Nuclear Medicine Laboratory of Shanghai University of Traditional Chinese Medicine, Shanghai, PR China).

### 2.10. Real-Time PCR

The expression levels of AK046177 and miR-134 were analyzed by real-time PCR in both brains and cell cultures after I/R or OGD/R. Total RNA was isolated from 30 mg cerebral cortical tissue or cells (1 × 10^6^/ml) seeded on 6-well plates with TRIzol Reagent (Invitrogen) [29], and reverse transcribed into cDNA using miScript Reverse Transcription Kit (Takara, China). Predesigned PCR primer/probes of miR-134 were obtained from GenePharma (Shanghai, China) for AK046177 and miRNA-134, with *β*-actin and U6 small nuclear RNA (U6) used as an internal control. Quantitative PCR was conducted as previously described using the TaqMan Assay Kit (Applied Biosystems) [30]. The relative expression levels of AK046177 and miRNA-134 were calculated using the 2^−ΔΔCT^ method [31].

### 2.11. Observation of Mitochondrial Morphology

Mitochondrial ultrastructure was analyzed using a transmission electron microscope (TEM) (Olympus, Tokyo, Japan). Primary cortical neurons were seeded in a 6-well plate and treated as described as above. After reperfusion, cells (9 × 10^5^/ml) were harvested and fixed in 2.5% (*v*/*v*) glutaraldehyde, then collected into a centrifuge tube using a scraper. Samples were fixed in osmic acid, dehydrated by the gradual addition of ethanol, and embedded in epoxy resin. Embedded samples were sectioned into ultraslices, stained with uranyl acetate and lead citrate, and then observed by TEM.

### 2.12. Measurement of Cell Respiration

Oxygen consumption rate (OCR) was measured using high-resolution respirometry in a 2-channel titration injection respirometer at 37°C (Oxygraph-2k, Oroboros, Innsbruck, Austria), as previously described by Pesta and Gnaiger [32]. Briefly, after reperfusion, cells (1 × 10^6^/ml) were harvested and placed in A and B pools with DMEM. Three readings were taken after the addition of each of the following mitochondrial inhibitors, prior to injection with the subsequent inhibitors. Inhibitors used were oligomycin (2 mg/ml), carbonyl cyanide chlorophenylhydrazine (CCCP; 10 mM), and rotenone (2 mM). OCR was automatically calculated and recorded by a sensor cartridge and Oroboros software.

### 2.13. Western Blot Analysis

Cerebral cortex tissues or cell cultures (1 × 10^6^/ml) were homogenized with lysis buffer and centrifuged at 12,000 g for 15 min. Forty micrograms of protein from each sample was separated on a SDS/10 and 15% polyacrylamide gel and then transferred to a polyvinylidene difluoride (PVDF) membrane (Millipore, IPVH00010, Bedford, MA, USA). After being blocked with 1% bovine serum albumin for 120 min at 20°C ± 2°C, membranes were incubated with anti-Nrf2 (1 : 1000; SAB4501984; Sigma-Aldrich), anti-Bcl-2 (1 : 1000; SAB4500003; Sigma-Aldrich), anti-Bax (1 : 1000; SAB4502546; Sigma-Aldrich), anti-caspase3 (1 : 500; ab32042; Abcam), anti-CREB (1 : 1000; SAB4500441; Sigma-Aldrich), anti-phospho-CREB (pSer^133^; 1 : 1000; C9102; Sigma-Aldrich), or anti-Nox4 (1 : 500; ab109225; Abcam) overnight at 4°C and washed with TBST (3 × 15 min). The corresponding horseradish peroxidase- (HRP-) conjugated secondary antibody was added and left for 50-60 min at 20°C ± 2°C, followed by washing with TBST (3 × 10 min). Protein bands were visualized using a chemiluminescence reagent (ECL kit; Amersham Corporation, Arlington Heights, CA, USA). The relative density of the protein bands was quantified by densitometry using an Image Acquiring and Analysis System (Leica Com., Germany). *β*-Actin was used to normalize protein loading. ERK1/2 phosphorylation was calculated as the ratio of normalized arbitrary units (a.u.) of (phosphorylated ERK1/2)/(total ERK1/2).

### 2.14. Determination of NADPH Oxidase Activity

NADPH oxidase activity was assayed using an Amplite™ Fluorime NADPH Assay Kit (AAT Bioquest, USA) according to the manufacturer's instructions. Primary cortical neurons were seeded in 24-well plates and treated. Rats were treated in vivo as described above. Cells (4 × 10^5^/ml) and tissues were lysed or homogenized, then centrifuged. The supernatant and NADPH oxidase reaction mixture were incubated at 37°C for 2 h. The increase in fluorescence was monitored using a microplate reader (BioTek Synergy H4, USA) at excitation and emission wavelengths of 540 and 590 nm, respectively.

### 2.15. Statistical Analysis

Data are presented as mean ± standard deviation (SD). The statistical analysis of the results was performed via one-way analysis of variance (ANOVA) followed by Student's *t*-test. All analyses were performed using SPSS 10.0 software (SPSS, Inc., San Rafael, CA, USA). Probabilities lower than 5% (*P* < 0.05) were considered statistically significant.

## 3. Results

### 3.1. Effect of SYB on Neurological Deficit Score, Infarct Volume, and Motor Function

As shown in Figures 1(b)–1(e), I/R significantly enhanced neurological deficit score and infarcted area and decreased motor scores compared to shams (*P* < 0.05 and *P* < 0.01). Intraventricular injection of AK046177 siRNA or intravenous injection of SYB was able to significantly decrease neurological deficit scores and infarct volume and enhance motor scores (*P* < 0.05 and *P* < 0.01, respectively). In addition, miR-134 agomir was capable of reversing the improvement in neurological deficit score, infarct volume, and motor scores of SYB and AK046177 siRNA (*P* < 0.05 and *P* < 0.01, respectively).

### 3.2. Effect of SYB on Cell Viability and Apoptosis

**Figure 2:**
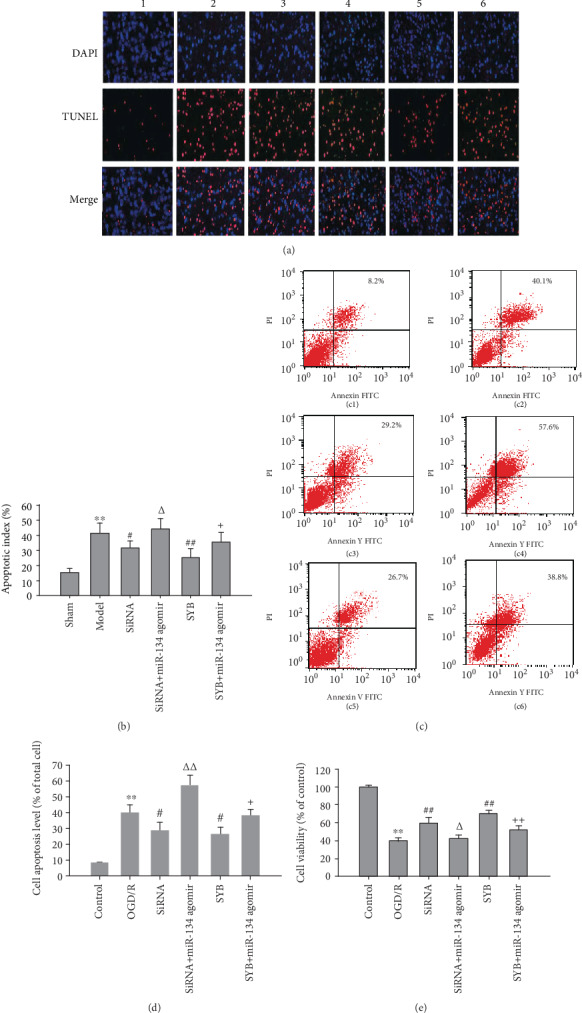
Effect of safflor yellow B on TUNEL-positive cells, cell viability, and apoptosis. Rats were divided into six groups: sham, ischemia/reperfusion (I/R), AK046177 siRNA, AK046177 siRNA+miR-134 agomir, SYB, and SYB+miR-134 agomir. (a) Representative images showing TUNEL-positive cells of cerebral cortex in different groups (×200 magnification; 1, 2, 3, 4, 5, and 6 represent sham, ischemia/reperfusion (I/R), AK046177 siRNA, AK046177 siRNA+miR-134 agomir, SYB, and SYB+miR-134 agomir, respectively). (b) Apoptotic (TUNEL-positive) cells were detected, AI = (number of apoptotic cells/total cell number counted) × 100%(*n* = 3). Primary fetal cortical cells were seeded in 96-well and 6-well plates and divided into six groups: control, OGD/R, AK046177 siRNA, AK046177 siRNA+miR-134 agomir, SYB, and SYB+miR-134 agomir. Apart from the control group, all cells in the other groups were cultured in glucose-free DMEM and hypoxic conditions (1% O_2_/94% N_2_/5% CO_2_) at 37°C for 4 h. Thereafter, all groups' media were replaced with normal DMEM, and culturing continued for 20 h of reoxygenation under normoxic conditions (95% air/5% CO_2_). The cells were pretreated with SYB, AK046177, and miR-134 agomir before being exposed to OGD/R. Cell viability was detected by the MTT method. Cell apoptosis was analyzed using flow cytometry. (c1)–(c6) represent control, OGD/R, AK046177 siRNA, AK046177 siRNA+miR-134 agomir, SYB, and SYB+miR-134 agomir, respectively. (d) represents cell apoptosis (*n* = 3). (e) represents cell viability (*n* = 8). Data are presented as mean ± SD. One-way ANOVA test was used to determine statistical significance. ^∗∗^*P* < 0.01*vs.* the sham group or the control group, ^#^*P* < 0.05 or ^##^*P* < 0.01*vs.* the I/R group or the OGD/R group, ^Δ^*P* < 0.05 or ^ΔΔ^*P* < 0.01*vs.* the AK046177 siRNA group, and ^+^*P* < 0.05 or ^++^*P* < 0.01*vs.* the SYB group.

I/R and OGD/R produced significant increases in the number of apoptotic cells in vivo (*P* < 0.01; Figures 2(a), 2(b), 2(c1), 2(c2), and 2(d)), as well as significant reductions in cell viability in vitro (*P* < 0.01; Figure 2(e)). This effect was significantly alleviated by AK046177 siRNA and SYB (*P* < 0.05 and *P* < 0.01, respectively; Figures 2(a), 2(b), 2(c3), 2(c5), 2(d), and 2(e)). miR-134 agomir inhibited the protective effect of AK046177 siRNA and SYB and then aggravated I/R- and OGD/R-mediated cell death and damage (*P* < 0.05 and *P* < 0.01, respectively; Figures 2(a), 2(b), 2(c4), 2(c6), 2(d), and 2(e)).

### 3.3. Effect of SYB on the Intracellular Expression of AK046177 and miR-134 and cAMP Content

**Figure 3:**
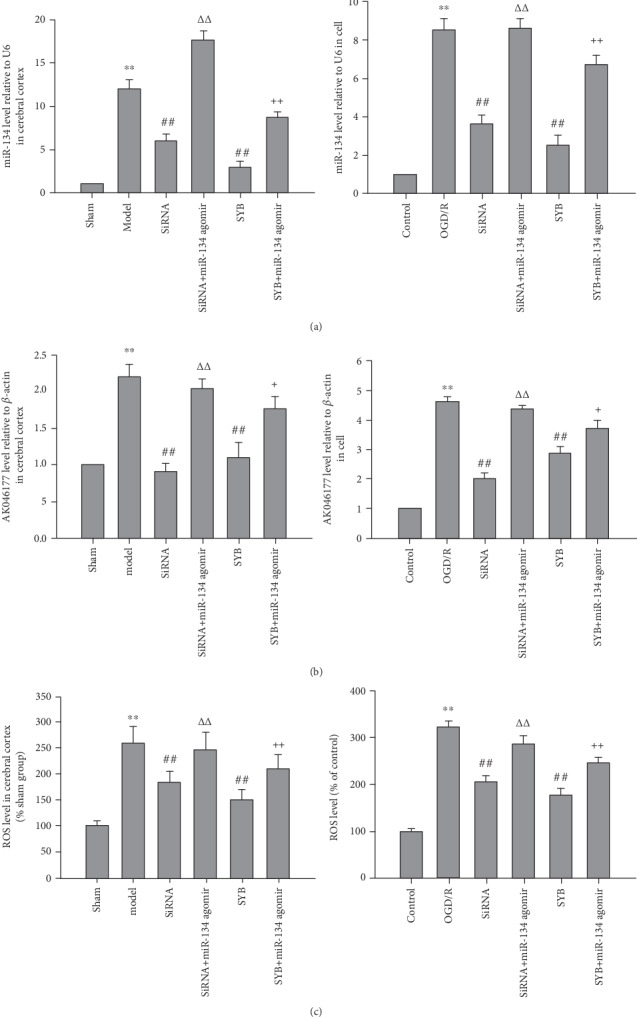
Effect of safflor yellow B on ROS level and the expression of AK046177 and miR-134. Rats and primary fetal cortical cells were used to establish the I/R model and the OGD/R model, respectively. After I/R or OGD/R for 24 h, the total RNA of every group was isolated and then reverse transcribed into cDNA. The expression levels of AK046177 and miR-134 were measured by real-time PCR. (a1), (a2), (b1), and (b2) represent miR-134 and AK046177 expression levels in the cerebral cortex and primary fetal cortical cells, respectively. ROS generation was measured according to the procedure of the assay kit provided by the manufacturer. (c1) and (c2) represent ROS levels in the cerebral cortex and the primary fetal cortical cells, respectively. Data are presented as mean ± SD (*n* = 8 in tissues; *n* = 3 in cells). One-way ANOVA test was used to determine statistical significance. ^∗∗^*P* < 0.01*vs.* the sham group or the control group, ^##^*P* < 0.01*vs.* the I/R group or the OGD/R group, ^ΔΔ^*P* < 0.01*vs.* the AK046177 siRNA group, and ^+^*P* < 0.05 or ^++^*P* < 0.01*vs.* the SYB group.

As shown in Figures 3(a1), 3(a2), 3(b1), and 3(b2), I/R and OGD/R significantly increased the expression levels of *AK*046177 and miR-134 (*P* < 0.01), which were decreased by the addition of AK046177 siRNA (*P* < 0.01). SYB effectively attenuated I/R and OGD/R induced increases in the expressions of *AK*046177 and miR-134 (*P* < 0.01), while the inclusion of miR-134 agomir reversed the SYB-induced attenuation (*P* < 0.05 and *P* < 0.01). Figures 1(f) and 1(g) demonstrate the significant reduction in intracellular cAMP levels caused by I/R and OGD/R; these reductions were further aggravated by miR-134 agomir (*P* < 0.05 and *P* < 0.01, respectively). Following treatment with AK046177 siRNA and SYB, intracellular cAMP levels rose significantly in both the cerebral cortex and the primary cultures (*P* < 0.05 and *P* < 0.01). miR-134 agomir significantly reduced the SYB- and AK046177 siRNA-induced increase in intracellular cAMP levels (*P* < 0.05).

### 3.4. Effect of SYB on SOD and GPx Activities and MDA Levels

**Figure 4:**
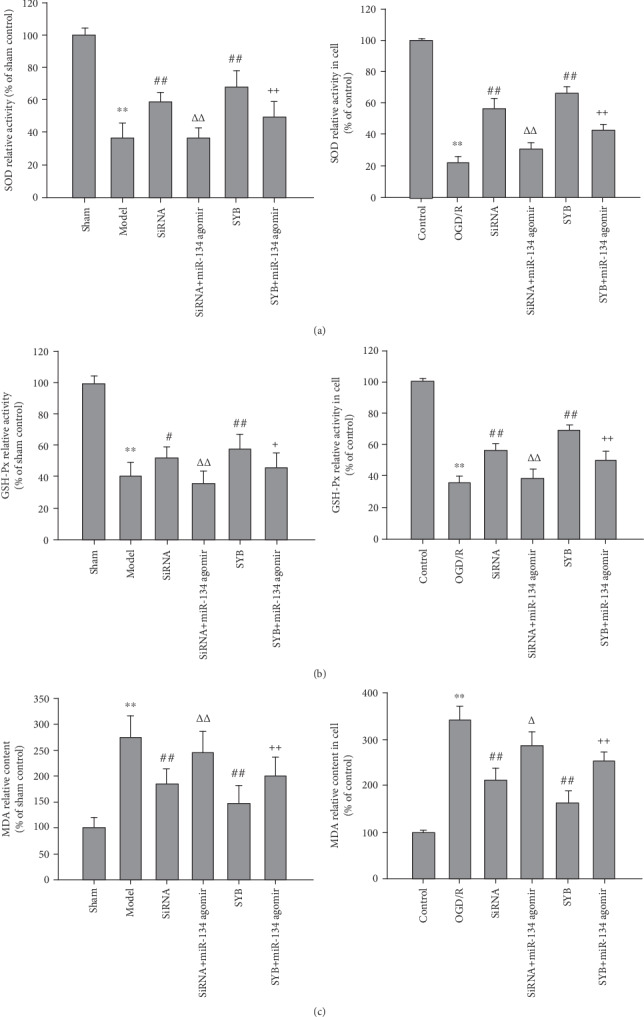
Effect of safflor yellow B on antioxidant enzyme activity and MDA level. Rats and primary fetal cortical cells were used to establish the I/R model and the OGD/R model, respectively. After I/R for 24 h, the cerebral cortex and cells from different groups were collected. The activities of SOD and GSH-Px and the MDA levels in tissues and cells were determined with the spectrophotometrical method according to the procedure described by the assay kit. (a1), (b1), and (c1) represent the activities of SOD and GSH-Px and the MDA level in cerebral cortex tissue. (a2), (b2), and (c2) represent the activities of SOD and GSH-Px and the MDA level in cells. Data are presented as mean ± SD (*n* = 8 in brain tissues, or *n* = 3 in cells). One-way ANOVA test was used to determine statistical significance. ^∗∗^*P* < 0.01*vs.* the sham group or the control group, ^#^*P* < 0.05 or ^##^*P* < 0.01*vs.* the I/R group or the OGD/R group, ^Δ^*P* < 0.05 or ^ΔΔ^*P* < 0.01*vs.* the AK046177 siRNA group, and ^+^*P* < 0.05 or ^++^*P* < 0.01*vs.* the SYB group.

As shown in Figures 4(a1), 4(a2), 4(b1), and 4(b2), the activities of SOD and GPx following I/R and OGD/R were reduced (*P* < 0.01), and MDA levels were increased (*P* < 0.01; Figures 4(c1) and 4(c2)) relative to those of the control group. Compared to the I/R or OGD/R groups, SYB significantly increased the activities of SOD and GPx in vitro and in vivo (*P* < 0.01). AK046177 siRNA promoted GPx activity in cells in a manner greater than the effect on SOD activity in vitro and in vivo and GPx activity in vitro (*P* < 0.01). SYB and AK046177 siRNA caused a significant reduction in MDA levels (*P* < 0.01). The effects of SYB and AK046177 siRNA were significantly suppressed by miR-134 agomir (*P* < 0.05 and *P* < 0.01, respectively).

### 3.5. Effect of SYB on NADPH Oxidase Activity, Nox4 Expression, and ROS Generation

**Figure 5:**
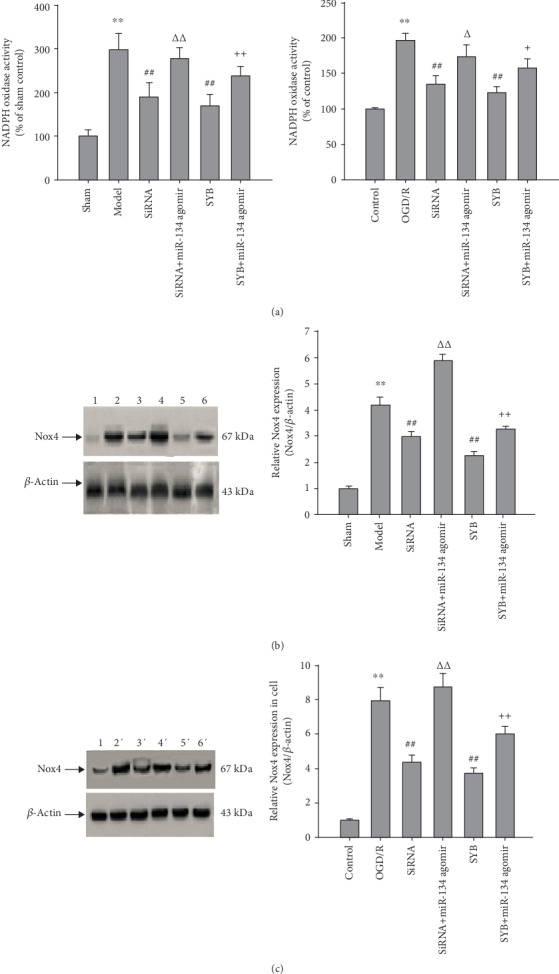
Effect of safflor yellow B on NADPH oxidase activity and Nox4 expression. Rats and primary fetal cortical cells were used to establish the I/R model and the OGD/R model, respectively. After I/R for 24 h, the cerebral cortex and cells from different groups were collected. NADPH oxidase activity and Nox4 expression was observed using Western blot analysis. (a1) and (a2) represent the NADPH oxidase activity of the cerebral cortex and the primary fetal cortical cells, respectively. (b1) Representative western blots of the cerebral cortex are shown (1, 2, 3, 4, 5, and 6 represent sham, ischemia/reperfusion (I/R), AK046177 siRNA, AK046177 siRNA+miR-134 agomir, SYB, and SYB+miR-134 agomir, respectively). (b2) represents Nox4 expression in the cerebral cortex. (c1) Representative western blots of primary fetal cortical cells are shown (1′, 2′, 3′, 4′, 5′, and 6′ represent control, OGD/R, AK046177 siRNA, AK046177 siRNA+miR-134 agomir, SYB, and SYB+miR-134 agomir, respectively). (c2) represent Nox4 expression in cells. Data are presented as mean ± SD (*n* = 8 in brain tissues, or *n* = 3 in cells). One-way ANOVA test was used to determine statistical significance. ^∗∗^*P* < 0.01*vs*. the sham group or the control group, ^##^*P* < 0.01*vs*. the I/R group or the OGD/R group, ^Δ^*P* < 0.05 or ^ΔΔ^*P* < 0.01*vs.* the AK046177 siRNA group, and ^+^*P* < 0.05 or ^++^*P* < 0.01*vs.* the SYB group.

As shown in Figures 5(a1) and 5(a2), compared to the control group, I/R and OGD/R significantly increased NADPH oxidase activity (*P* < 0.01). Compared to the I/R or OGD/R groups, AK046177 siRNA and SYB were capable of significantly abolishing NADPH oxidase activity (*P* < 0.01). After treatment with miR-134 agomir, NADPH oxidase activity in the cerebral cortex and primary neuronal cultures showed a significant increase relative to the SYB and AK046177 siRNA groups (*P* < 0.05 and *P* < 0.01, respectively).

Nox4 directly reflects NADPH oxidase activity. Our data showed that I/R and OGD/R significantly promoted Nox4 expression relative to the sham and control groups (*P* < 0.01). Both SYB and AK046177 siRNA were able to significantly suppress Nox4 expression, an effect reversed by miR-134 agomir (*P* < 0.01 and *P* < 0.05; Figures 5(b1), 5(b2), 5(c1), and 5(c2)).

ROS levels in cells exposed to OGD/R and cerebral cortex of rats induced by I/R were higher than those of the control group and the sham group (*P* < 0.01; Figures 3(c1) and 3(c2)). This effect was significantly mitigated by AK046177 siRNA and SYB, which also decreased ROS generation relative to the OGD/R group and the sham group (*P* < 0.01). The inhibitory effects of SYB and AK046177 siRNA on ROS generation were significantly ameliorated by miR-134 agomir in vitro (*P* < 0.01).

### 3.6. Effect of SYB on the Expressions of Nrf2, CREB, pCREB, Caspase3, Bcl-2, and Bax

**Figure 6:**
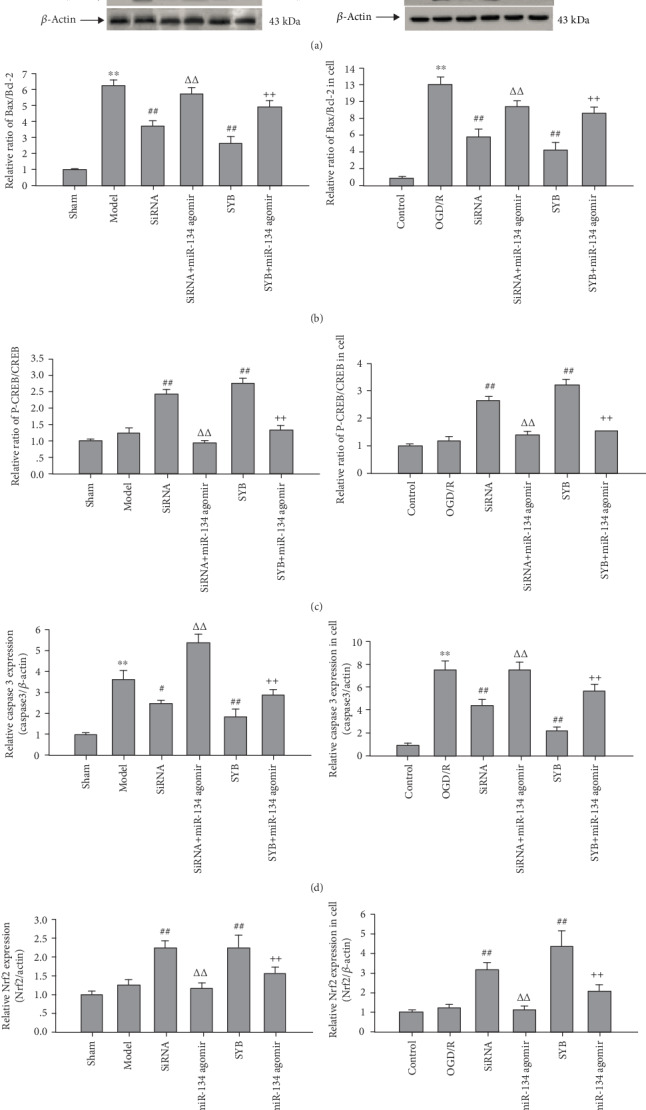
Effect of safflor yellow B on target protein expression. Rats and primary fetal cortical cells were used to establish the I/R model and the OGD/R model, respectively. After I/R for 24 h, total protein in the cerebral cortex and cells from different groups were extracted and measured. Thirty micrograms of protein was loaded per lane and separated by SDS-PAGE and transferred to a PVDF membrane. After incubation with secondary antibodies, the expression levels of CREB, pCREB, Bcl-2, Bax, and Nrf2 were visualized using the chemiluminescence method. (a1) and (a2) represent Western blots (1, 2, 3, 4, 5, and 6 represent sham, ischemia/reperfusion (I/R), AK046177 siRNA, AK046177 siRNA+miR-134 agomir, SYB, and SYB+miR-134 agomir, respectively; 1′, 2′, 3′, 4′, 5′, and 6′ represent control, OGD/R, AK046177 siRNA, AK046177 siRNA+miR-134 agomir, SYB, and SYB+miR-134 agomir, respectively). (b1), (c1), (d1), and (e1) represent the relative ratio of Bax/Bcl-2 and pCREB/CREB, caspase3, and Nrf2 expression in the cerebral cortex, respectively. (b2), (c2), (d2), and (e2) represent the relative ratio of Bax/Bcl-2 and pCREB/CREB, caspase3, and Nrf2 expression in cells, respectively. Data are presented as mean ± SD (*n* = 3). One-way ANOVA test was used to determine statistical significance. ^∗∗^*P* < 0.01*vs.* the sham group or the control group, ^#^*P* < 0.05 or ^##^*P* < 0.01*vs.* the I/R group or the OGD/R group, ^ΔΔ^*P* < 0.01*vs.* the AK046177 siRNA group, and ^++^*P* < 0.01*vs.* the SYB group.

As seen in Figures 6(a1)–6(e2), the ratio of Bax/Bcl-2 and caspase3 expression in the I/R and OGD/R groups were higher than those in the sham or control groups (*P* < 0.01). AK046177 siRNA and SYB significantly increased Nrf2 expression and the ratio of pCREB/CREB and decreased caspase3 expression and the ratio of Bax/Bcl-2, relative to the I/R and OGD/R groups (*P* < 0.01 and *P* < 0.05). miR-134 agomir reversed these effects, and increased the ratio of Bax/Bcl-2 and caspase3 expression, and reduced the ratio of pCREB/CREB and Nrf2 expression, relative to the AK046177 siRNA and SYB groups (*P* < 0.01 and *P* < 0.05).

### 3.7. Effect of SYB on Mitochondrial Structure and Cell Respiration

**Figure 7:**
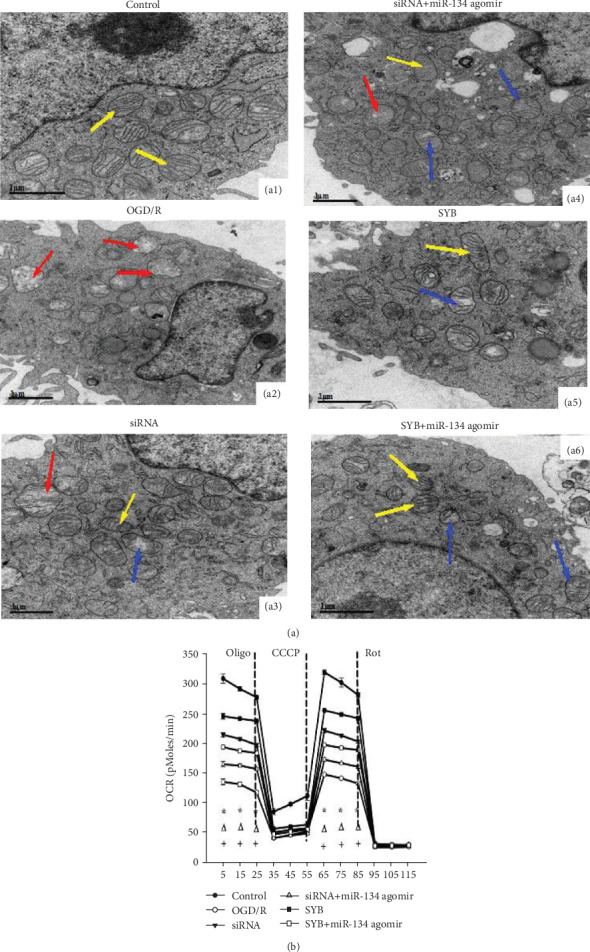
Effect of safflor yellow B on mitochondrial structure and cell respiration. Primary fetal cortical cells were seeded in 96-well and 6-well plates and divided into six groups: control, OGD/R, AK046177 siRNA, AK046177 siRNA+miR-134 agomir, SYB, and SYB+miR-134 agomir. The cells were pretreated with the drugs including SYB, AK046177 siRNA, and miR-134 agomir before being dealing with OGD/R. (a1–a6) Mitochondrial structure was evaluated using a transmission electron microscope (×20,000 magnification, bar 1 *μ*m) and represent the mitochondria structure of the control group, the OGD/R group, AK046177 siRNA, AK046177 siRNA+miR-134 agomir, SYB, and SYB+miR-134 agomir, respectively. Transmission electron microscope shows normal mitochondrial structure (yellow solid line arrows), and pathological mitochondria with irregular shapes and swollen (red solid line arrow) and vesicular mitochondrial clusters (blue solid line arrow). Cellular oxygen consumption rate (OCR) was measured using an Oxygraph-2k system (*n* = 3 experiments per condition). Data are presented as mean ± SD (*n* = 3). One-way ANOVA test was used to determine statistical significance. ^∗^*P* < 0.01*vs.* the control group, ^#^*P* < 0.01*vs.* the OGD/R group, ^Δ^*P* < 0.01*vs.* the AK046177 siRNA group, ^+^*P* < 0.01*vs.* the SYB group.

In the control group, the mitochondria were diffused throughout the cell, with an oval or rod-like shape and normal structure (Figure 7(a1)). In OGD/R-treated cells, mitochondria exhibited pathological changes including irregular and swollen shapes, mitochondrial ridge fault, and vesicular mitochondrial clusters, especially adjacent to the cell nucleus (Figure 7(a2)). After treatment with SYB and AK046177 siRNA, an increased number of normal mitochondria were detected (Figures 7(a3) and 7(a5)). miR-134 agomir in addition to SYB and AK046177 siRNA resulted in more irregular and swollen shapes, more mitochondrial cristae fracturing, and severe vacuolization within mitochondria compared to the SYB group (Figures 7(a4) and 7(a6)). Moreover, AK046177 siRNA and SYB were capable of effectively alleviating mitochondrial pathology induced by miR-134 agomir and OGD/R.

OGD/R markedly influenced cellular respiration and decreased the OCR (Figure 7(b)). The OCR in all groups was significantly reduced after treatment with oligomycin. Cells normally increase OCR in response to CCCP in order to maintain the proton gradient and mitochondrial function. However, cells exposed to OGD/R had a smaller increase in OCR after CCCP treatment compared to controls, suggesting that these cells have impaired respiratory capacity. Administration of rotenone, a complex I inhibitor, also inhibited OCR. These data suggest that OGD/R results in the loss of mitochondrial oxidative phosphorylation (OXPHOS) function in primary neurons. Furthermore, AK046177 siRNA and SYB enhanced OCR after CCCP treatment relative to the OGD/R group, suggesting that AK046177 siRNA and SYB were able to improve mitochondrial respiratory capacity. Under OGD/R conditions, miR-134 agomir significantly reduced the OCR increase induced by AK046177 siRNA and SYB and amplified OGD/R-mediated cell injury, resulting in much weaker respiratory capacity.

## 4. Discussion

lncRNAs may act to upregulate biological processes in various disease states by directly or indirectly interacting with mRNA of the target gene, leading to the modulation of apoptosis and invasion and modification of chromatin [18–20].

MicroRNAs are a type of small noncoding RNA involved in various diseases via their interaction with the mRNA of target genes, which leads to the destabilization and degradation of mRNA [33–35]. Di et al. have shown that a variety of miRNAs are involved in cerebral I/R injury [36]. The brain-specific miR-134 was reported to be differentially expressed in tissue subjected to MCAO and I/R injury [37]. Previous studies had shown that lncRNAs influenced ischemia-mediated tissue damage via regulation of microRNAs [38–40]. In the present study, we demonstrated that I/R and OGD/R increased the expression levels of miR-134 in the cerebral cortex or primary neuronal cultures and resulted in neuronal damage accompanied by the upregulation of AK046177. Inhibition of AK046177 expression could significantly reduce the expression of miR-134, thereby alleviating I/R- or OGD/R-mediated neuronal injury.

Wang et al.'s studies have shown that SYB was able to effectively inhibit ischemia-induced brain injury [24]. However, the relationship between miR-134 or AK046177 and SYB in cerebral ischemia was unknown. These data confirm that SYB decreases the expression levels of miR-134 and AK046177 and reduces I/R- and OGD/R-induced damage.

Cyclic AMP is an important second messenger involved in many biochemical processes through its ability to regulate protein kinase activity. It is involved in diverse processes such as platelet activation, thrombus formation, inflammatory response, and oxidative stress responses [41–43]. Elevation of intracellular cAMP levels can change the antioxidant capacity of the cells by regulating the phosphorylation of CREB [44]. Thus, CREB has important influences in various diseases [45, 46]. Substantial evidence indicates that CREB plays critical roles in the neuronal responses to ischemia, such that activation and overexpression of CREB significantly reduces ischemia-mediated brain injury and increases BDNF and Bcl-2 expression [15, 47]. Mabuchi et al. and Huang et al. showed that miR-134 directly targets the 3′-UTR of CREB mRNA, influences the translation and phosphorylation of CREB, and suppresses BDNF and Bcl-2 expression, leading to apoptosis [47]. Conversely, the activation of miR-134 suppresses the expression of CREB and aggravates tissue or cell injury.

Nrf2 is an important transcription factor that can alleviate oxidative stress-mediated cellular damage by activating the transcription of antioxidant enzymes. Nguyen et al. demonstrated the potential of Nrf2-mediated transcription to protect the brain from neurodegeneration resulting from oxidative stress [48]. The phosphorylation of CREB also enhances the binding of Nrf2 to its DNA response element [49, 50]. In the present study, we found that I/R and OGD/R induced overexpression of miR-134 and AK046177 and reduced intracellular cAMP levels, which in turn suppressed the expression and phosphorylation of CREB. The result was a further downregulation of Nrf2 and upregulation of Bax and caspase3, eventually leading to oxidative damage and apoptosis.

Inhibition of AK046177 increased cAMP synthesis and promoted the expression and phosphorylation of CREB, and then stimulated Nrf2 activation and effectively attenuated I/R- or OGD/R-mediated injury by the downregulation of miR-134. Previous studies have shown that the neuroprotective effect of SYB is related to increasing antioxidative enzyme activities, decreasing free radical generation, and regulating Bcl-2/Bax signaling [24], each of which was confirmed in the present study. Moreover, our data demonstrated that SYB also improved cAMP synthesis, and then enhanced the ratio of pCREB/CREB and activated Nrf2 expression via the suppression of overexpressions of AK046177 and miR-134 induced by I/R or OGD/R.

Increased mitochondrial oxidative stress induces mitochondrial dysfunction, which has been linked to a variety of diseases including cardiovascular disease [51, 52]. Mitochondrial dysfunction increases ROS due to electrons escaping from the mitochondrial respiratory chain and interacting with oxygen molecules. ROS destroy mitochondrial membranes and influence cell respiration, facilitate the release of cytochrome C, and promote activation of caspase3, ultimately leading to apoptosis [53, 54].

Nrf2 is a prominent player in supporting the structural and functional integrity of the mitochondria under conditions of stress, and thus plays a crucial role in the maintenance of cellular redox homeostasis. It regulates the mitochondrial production of ROS and NADPH oxidase [55]. In the present study, we discovered that I/R and OGD/R caused mitochondrial structural damage, such as an increase in mitochondrial volume and the formation of fractured cristae, and increased NADPH oxidase activity. This damage resulted in mitochondrial dysfunction, demonstrated by lower OCR and ROS generation. Both AK046177 siRNA and SYB were able to increase the number of healthy mitochondria, dramatically enhance cellular respiratory function, and inhibit ROS production. However, miR-134 agomir remarkably increased ROS levels by blocking the mitochondrial respiratory chain and reducing the increased activity of antioxidant enzymes related to CREB binding, thereby boosting apoptosis.

## 5. Conclusion

In this study, SYB protected the brain from I/R-related injury by increasing intracellular cAMP levels, downregulating overexpressions of AK046177 and miR-134, and activating the CREB/Nrf2 pathway. The mechanisms regulating I/R injury are complicated, involving a variety of miRNAs and signaling pathways [35, 56]. We believed that these data only reflected one aspect of the event, and some important proteins may contribute to the understanding of the mechanisms involved in I/R-mediated neuronal injury.

## Data Availability

The data is available on request by asking directly the corresponding author by mail at ytwcy@163.com.

## Abbreviations

SYB: Safflor yellow B
ROS: Reactive oxygen species
CREB: Cyclic AMP (cAMP) response element-binding protein
Nrf2: Nuclear factor erythroid 2-related factor 2
AK046177: Long noncoding RNA046177
miR-134: MicroRNA-134
I/R: Ischemia/reperfusion
OGD/R: Oxygen-glucose deprivation
cAMP: Cyclic AMP
ARE: Antioxidant response elements
BDNF: Brain-derived neurotrophic factor
DMEM: Dulbecco's minimal essential medium
MCAO: Middle cerebral artery occlusion
SOD: Superoxide dismutase
GPx: Glutathione peroxidase
MDA: Malondialdehyde
HSYA: Hydroxysafflor yellow A
OCR: Oxygen consumption rate
CCCP: Carbonyl cyanide chlorophenylhydrazine
OXPHOS: Oxidative phosphorylation
MTT: 3-(4,5-dimethyithiazol-2-yl)-2,5-diphenyl-tetrazolium bromide.
